# Persistent perineal sinus following proctocolectomy in the inflammatory bowel disease patient

**DOI:** 10.1002/jgh3.12983

**Published:** 2023-10-27

**Authors:** Sam D. Papasotiriou, Gregory A. Dumanian, Scott A. Strong, Stephen B. Hanauer

**Affiliations:** ^1^ Northwestern University Chicago Illinois USA

**Keywords:** Crohn's colitis, inflammatory bowel disease clinical, persistent perineal sinus, ulcerative colitis

## Abstract

Prolonged perineal wound healing following proctocolectomy in patients with inflammatory bowel disease (IBD) is a frustrating result for the medical team and patients who were hoping for improved quality of life. Prolonged healing, which lasts more than 6 months following proctocolectomy, is termed persistent perineal sinus (PPS) and typically necessitates further surgical management. Healing of the PPS is difficult due to the resulting “dead space” following proctocolectomy, necessitating the need to fill the void with viable tissue in an area with anatomic constraints. Here we provide a narrative review and comprehensively address the incidence, pathogenesis, and clinical and operative management of a PPS in patients with IBD following proctocolectomy. Operative methods discussed include surgical debridement, flap closure of the perineum, omental flap closure, and gracilis muscle transposition. It is necessary to further investigate and establish a gold standard of care for these patients.

## Graphical abstract

A review on the latest medical literature pertaining to the persistent perineal sinus following proctocolectomy for the inflammatory bowel disease patient providing discussion of incidence, pathogenesis, and clinical and operative management.

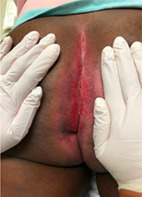

## Introduction

**Figure 1:**
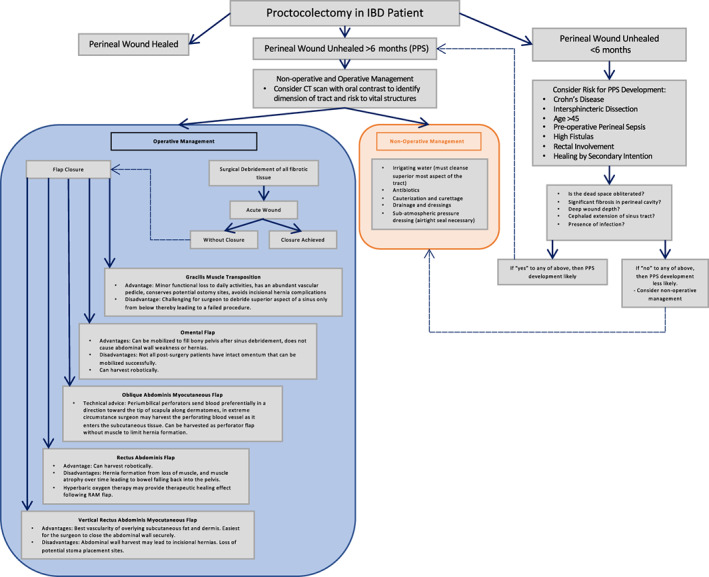
Algorithm for the diagnosis and management of persistent perineal sinus (PPS). CT, computed tomography; IBD, inflammatory bowel disease.

Poor perineal wound healing has been identified as a common postoperative complication for patients undergoing proctocolectomy for rectal cancer, familial adenomatous polyposis, and inflammatory bowel disease (IBD). Patients with IBD suffer prolonged post‐proctocolectomy perineal healing more frequently than patients treated for neoplasia,^1^ and this complication negatively impacts the intended post‐colectomy quality of life. Patients with poor wound healing commonly suffer from drainage, pain, and bleeding. An incompletely healed perineal wound that persists for more than 6 months following proctectomy is termed a persistent perineal sinus (PPS) and typically requires further surgical management.^2^ A PPS is occasionally further complicated by a cephalad extension, which appears as a tract of variable dimensions lined by granulation tissue and biofilm that extends cephalad into the pelvis, often occupying the presacral region. Historically, studies have reported the incidence of PPS development ranging from 25% to 56% following proctocolectomy for IBD.^3^, ^4^, ^5^ In this review, we discuss the incidence, pathogenesis, and clinical management of PPS in patients with IBD following proctectomy. Given the complicated nature of this disease, Figure 1 displays an algorithm created by Papasotiriou *et al*., which outlines the diagnosis and management of PPS.

## Pathogenesis and anatomy of PPS

**Figure 2:**
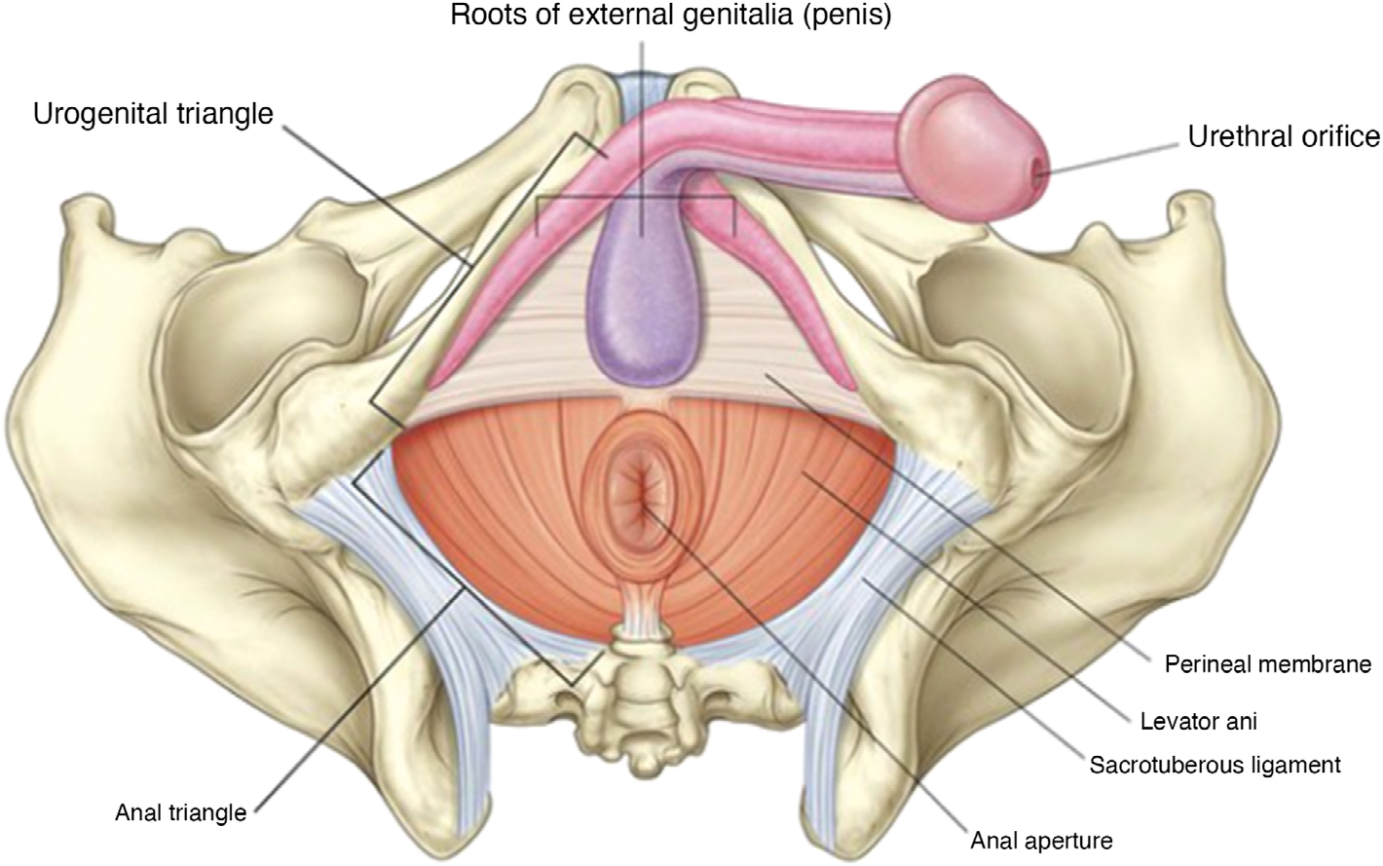
Male perineum.^6^

**Figure 3:**
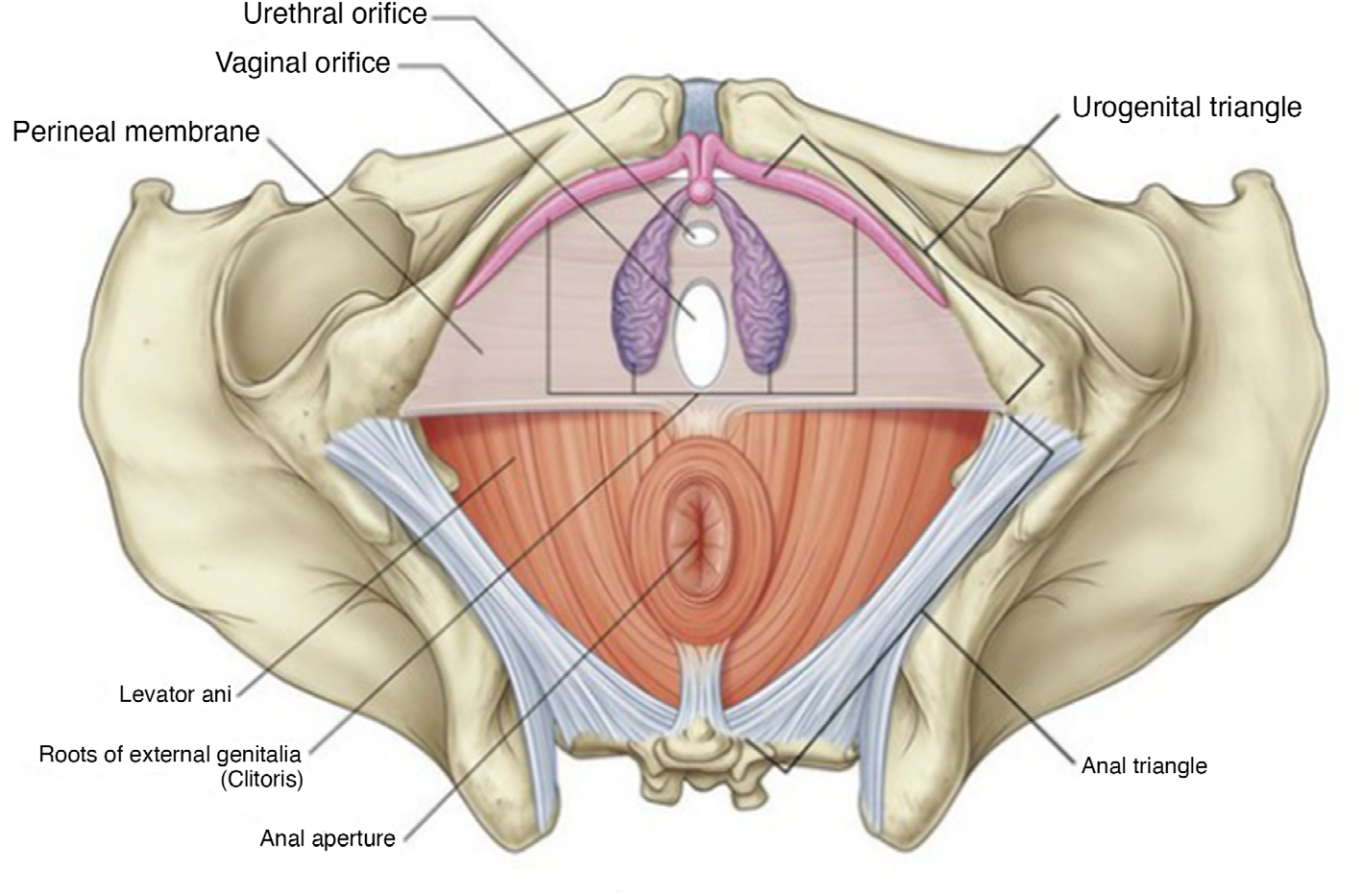
Female perineum.^6^

**Figure 4:**
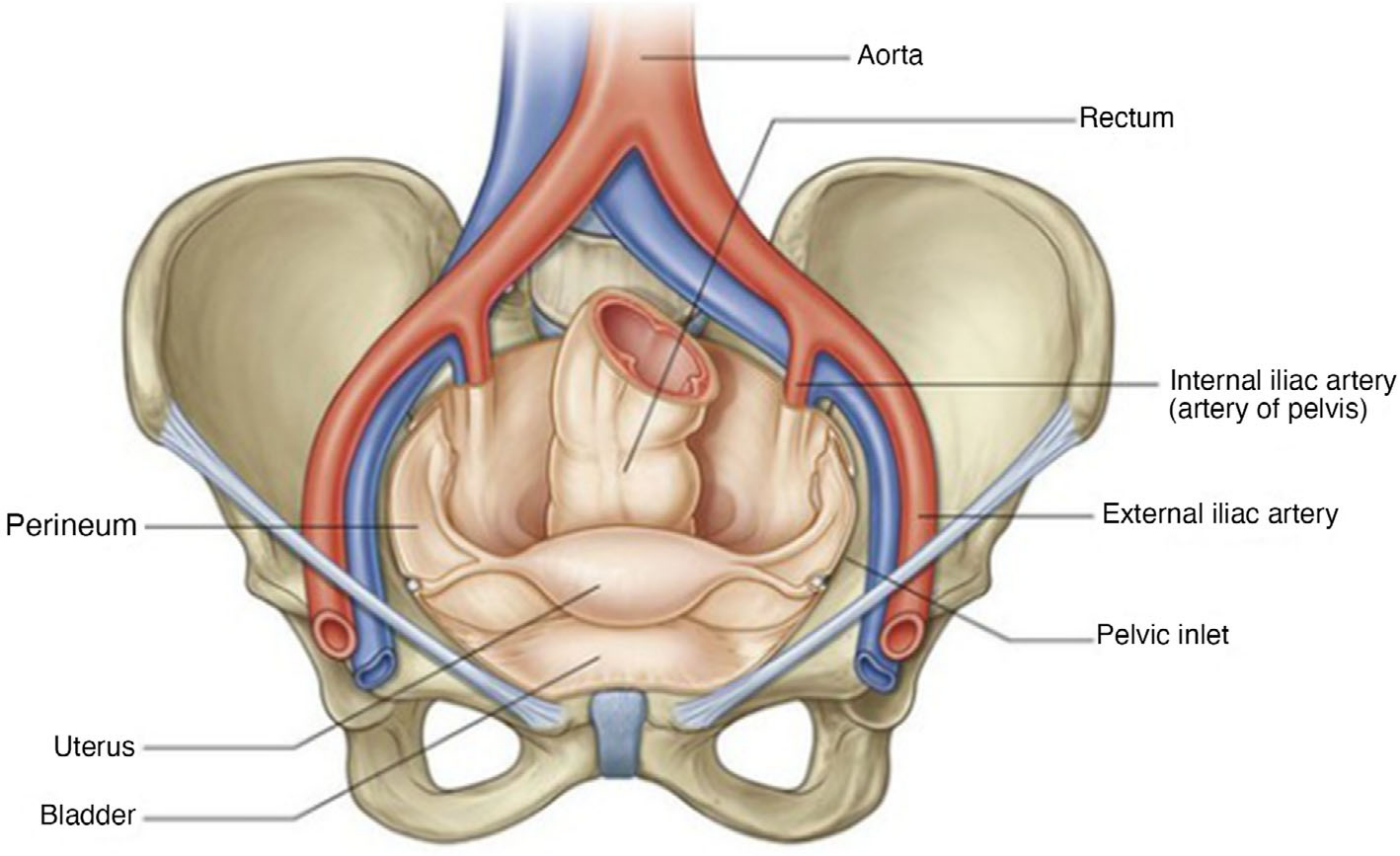
Pelvis cavity and perineum.^6^

The perineum is located at the caudal region of the trunk, medial to the thighs. The anatomic boundaries of the cavity consist of the pubic symphysis and urogenital organs anteriorly, the ischiopubic rami anterolaterally, ischial tuberosities laterally, sacrotuberous ligaments posterolaterally, the sacrum and coccyx posteriorly, and the pelvic peritoneum forming the superior boundary. Figures 2, 3, 4 show an anatomical representation of the perineum.

Surgical proctectomy involves the removal of the diseased rectum, resulting in a “dead space” that must heal “from inside out” to avoid postoperative complications such as a PPS. The anatomic constraints of the perineum challenge the surgeon to fill this void with viable tissue. The bones of the lateral and posterior pelvis are immobile, so filling of the empty space occurs through upward migration of the buttocks, posterior displacement of the urogenital structures, and downward relocation of the abdominal contents.^7^, ^8^ The unyielding bony pelvis resists the tendency for soft tissues to contract into an unfilled space. Furthermore, healing of the dead space may be complicated by various factors, including septic contamination during operation, poor hemostasis, and postoperative fluid collection in the cavity, risking bacterial growth, abscess formation, and local tissue ischemia.^8^

Healing of the perineal wound becomes challenging in the setting of fibrotic tissue, which decreases perfusion of the site resulting in reduced tissue nutrition and oxygenation, leading to the development of an ischemic wound comparable to chronic pleural cavity empyemas.^7^, ^8^ If the perineal wound fails to heal from inside outward, combined with any of the aforementioned risk factors, then a PPS likely develops.

## Incidence, risk factors, and prevention

The incidence of proctocolectomy for the treatment of IBD varies depending on the underlying diagnosis, and is reported as 12–20%^9^, ^10^ for failure of medical therapy of Crohn's disease (CD) and 5–20% for ulcerative colitis (UC) within 10 years of diagnosis.^11^ Unfortunately, PPS is a common postoperative complication of proctocolectomy and can be as troublesome and debilitating as the patient's previous disease state.^1^, ^8^, ^12^

The incidence of PPS as a post‐surgical morbidity in the IBD patient ranges from 3% to 70% at 6 months, and as high as 33% at 12 months.^13^, ^14^, ^15^, ^16^, ^17^ Proctectomy for IBD usually entails removal of the rectum and internal sphincter (intersphincteric dissection) while preserving muscles of the pelvic floor and external sphincter. CD sometimes requires a more extended resection with excision of the external sphincter muscles and perianal skin (extrasphincteric dissection), especially when it is necessary to remove all infected, fistulizing, malignant, or scarred tissue. This more extensive excision causes a higher incidence of perineal wound dehiscence and complications in patients with CD compared to those with UC.^18^ In the past, Corman *et al*. reported that 72% of patients with CD experienced an unhealed perineal wound 6 months after surgery compared to 56% of those with UC.^19^ In a study by Yamamoto *et al*., the records of 145 patients who underwent proctocolectomy from 1970 to 1997 for CD were reviewed; a PPS was reported in 23% of patients.^18^ In a more recent study by Beddy *et al*., PPS occurrence was reported in up to 25% of CD proctectomies.

In contrast, the surgical technique of proctectomy for UC generally results in a smaller wound because the plane of perineal dissection is limited to the intersphincteric space. The resulting wound can be primarily closed in multiple layers with less tension on the closure, leading to a reduced likelihood of PPS.^20^

Incomplete or delayed healing of the perineum following proctocolectomy for IBD has been attributed to several risk factors. The primary cause of a non‐healing sinus is surface bacteria along the wound tract, which are difficult to clean and remove. The long track complicates dressing changes and the removal of surface bacteria, especially at its most superior extent. Other causes include the retention of foreign bodies (e.g. drains, non‐absorbable sutures, swabs), sequestrum formation due to extension of infection into the sacrum, and recurrent neoplasm. Difficult excision leading to incomplete resection of the rectal mucosa may also contribute as a primary cause to sinus tract formation.^7^, ^18^ Yamamoto *et al*. noted additional risk factors associated with significantly greater risk of PPS, including older age (≥45 years), rectal involvement, fecal contamination, perineal sepsis, high fistula‐in‐ano or rectovaginal fistula, and extrasphincteric excision. In contrast to older studies, the authors did not find a statistically significant increased risk due to cigarette smoking, preoperative corticosteroid use, or sex, associated with PPS development.^18^ Although not statistically significant, it is important to consider that the prostate may interfere with the healing of the perineal wound due to a reduced ability to collapse the aforementioned dead space or void. A more recent 2017 retrospective study by Li *et al*. included 136 patients with CD who underwent proctocolectomy or proctectomy at the Cleveland Clinic between 1995 and 2012 and challenged previous attributions of risk factors associated with PPS following proctocolectomy. The above authors discussed the perineal wound outcome in four distinct categories: normal healing (<3 months), delayed healing (3–6 months), non‐healing (>6 months), and non‐healing with the development of a PPS. Of the 136 patients included in the study, 29 patients (21%) experienced non‐healing, with 9 of these patients (6.5%) developing a PPS. The study found no increased risk of poor perineal wound healing associated with preoperative smoking, age, sex, or corticosteroid use. Multivariate analysis suggested that only the presence of preoperative perineal sepsis was linked to the occurrence of PPS.^10^ Table 1 shows the number (*n*) of individuals with a risk factor, as discussed above, who developed a PPS.

**Table 1:** Risk factors for persistent perineal sinus in inflammatory bowel disease

Risk factor	Number *n* (%)^†^	*P*‐value	Study	Study year
CD	44/61 (72)	0.01	Corman *et al*.	1978
UC	50/90 (56)	0.01	Corman *et al*.	—
Age			Yamamoto *et al*.	1999
<25 years	14/40 (35)	0.006		
26–44 years	17/67 (26)			
>45 years	2/38 (6)			
Rectal involvement	32/118 (27)	0.02	Yamamoto *et al*.	—
Preoperative perineal	22/57 (39)	0.0005	Yamamoto *et al*.	—
Sepsis	17/57 (30)	0.001	Li *et al*.^‡^	2017
Fecal contamination	14/28 (50)	0.0003	Yamamoto *et al*.	—
High fistulas	14/39 (36)	0.04	Yamamoto *et al*.	—
Extrasphincteric dissection	28/82 (34)	0.0004	Yamamoto *et al*.	—
Healing by secondary intention	12/38 (32)	0.02	Li *et al*.^‡^	—

^†^ Number of individuals with the risk factor who developed a persistent perineal sinus divided by total number of individuals with the risk factor.

^‡^ Study authors describe these risk factors effect on poor perineal wound healing with a small cohort of chronic presacral sinus patients.

CD, Crohn's disease; UC, ulcerative colitis.

Prevention of a PPS is multifactorial and dependent on the ability to identify the aforementioned risk factors and mitigate operative risk for development. Consideration must be to whether proctocolectomy is performed in the CD *versus* the UC patient. In all patients, any modifiable preoperative risk factors should be corrected, and the surgeon should avoid fecal spillage and incomplete excision of the involved bowel at the time of surgery. Moreover, it may be beneficial to fill the empty pelvis with a flap of biologic tissue (e.g. muscle, omentum) at the time of surgery to avoid development of a perineal sinus in selected high‐risk patients. Patients with CD and perineal sepsis may benefit from at least 3–6 months of fecal diversion before undergoing proctectomy. Interdisciplinary collaboration is commonly required to provide the care needed to prevent PPS formation.

## Clinical presentation

**Figure 5:**
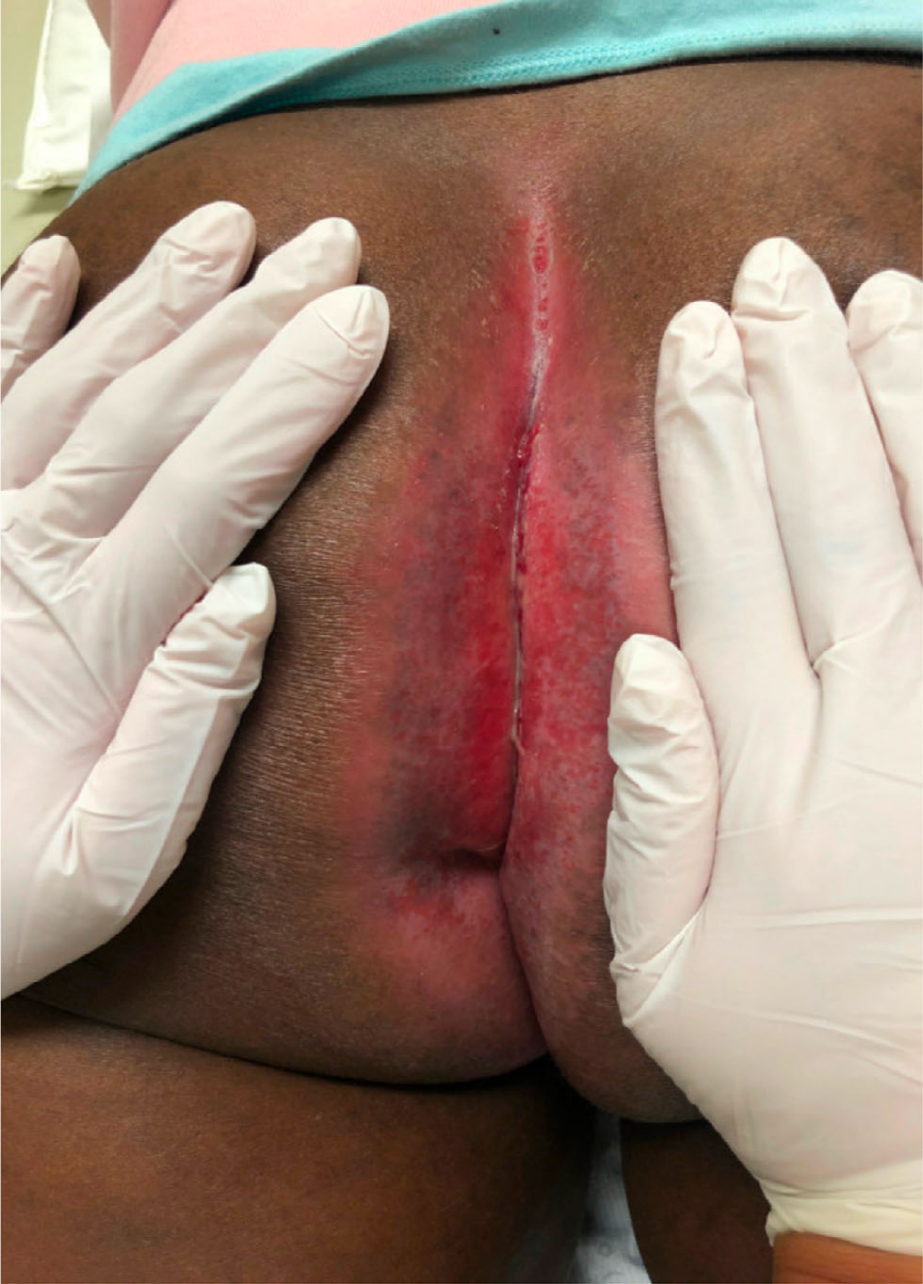
Persistent perineal sinus following proctocolectomy in inflammatory bowel disease patient.

Following proctocolectomy, postoperative perineal wound closure is considered when the skin is closed and dry with no seepage. Patients suffering from a PPS present, 6 months following operation, with drainage, skin ulceration, or wound infection.^2^ In addition to drainage, ulceration, and wound infection, a patient may also have occasional bleeding or pain in the perineal area especially while sitting.^20^ On examination of the PPS, a fibrous tract covered with granulation tissue and an occasional external opening will be observed. Observations of the defect size, skin and tissue condition (e.g. abscess, erythema, fibrosis, inflammation), and presence of infection should be noted. Figure 5 shows a patient with IBD who underwent proctocolectomy and subsequently developed a PPS. It is necessary to determine whether the PPS has signs of primary causes (retained foreign body, sequestrum formation due to extension of infection into the sacrum, or recurrent neoplasm)^7^, ^8^, ^18^ via computed tomography (CT)/magnetic resonance imaging (MRI), dedicated small‐bowel imaging, or sinography, while it is also recommended that any discharge be swabbed for bacterial culture in order to assess for secondary infection. Furthermore, biopsy should be considered in selected cases because of the risk of chronic unhealed wounds representing malignancy.^8^, ^9^

## Management

Multiple factors dictate the type of management required, such as superficial *versus* deep wound depth, simple PPS *versus* PPS associated with a cephalad tract extension, presence of infection, and the amount of suppuration and fibrosis present. The walls of the cavity may become rigid in the presence of suppuration or significant fibrosis, resulting in a cavity that becomes less likely to collapse and close.^7^ Proper healing of the perineum requires that any “dead space” is completely obliterated, and depending on whether a non‐operative or operative approach is required, management of PPS should be navigated with the intent of eliminating or addressing all nonviable margins.

### Non‐operative

Conservative treatment of a PPS is typically trialed first and may involve various or multiple methods, including irrigation, antibiotics, cauterization, curettage, drainage, dressings, irrigating water, and sub‐atmospheric‐pressure dressing.^8^, ^9^

Local wound care and application of topical metronidazole ointment on the sinus has been reported to decrease perineal pain but does not close the sinus. Fibrin glue has also been unsuccessful at closing the sinus.^8^ Curettage may be beneficial for the superficial PPS to remove the fibrous lining of the track and establish “granulating margins” to promote healing of the cavity.^7^ A pulsating water irrigation works to clean bacteria from the surface of sinus tracts, but cleansing of the superior‐most aspect of the tract is essential for success.

Use of sub‐atmospheric‐pressure dressing is another noninvasive approach to PPS closure to reduce the size of the cavity, decrease bacterial cell counts, and stimulate the formation of granulation tissue; these factors aid in the contraction and migration of tissue and resultant closure of the sinus. In addition, this method removes wound fluid and has been shown to increase skin perfusion, ultimately increasing oxygenation of the sinus. An airtight seal is necessary when applying a sub‐atmospheric‐pressure device and convexities of the buttocks and exudate production may interfere with achieving this seal.^21^ Negative‐pressure dressings are contraindicated in patients with malignancy or nonviable tissue in the wound.

Conservative treatment may be required for months or years in order to completely heal the PPS.^21^

### Operative

The outcome for operative treatment is variable (Table 2), precluding “definitive” (evidence‐based) treatment guidelines. Literature pertaining to the surgical management of PPS in IBD patients is limited by the number of studies, number of patients included, and follow‐up period. Operative options can be categorized into surgical debridement with or without reclosure, muscle/myocutaneous flaps, and omental flap.

**Table 2:** Surgical treatments of persistent presacral sinus published in the past 45 years

Surgical method	Author	Publication year	No. of patients, *n*	IBD type	Complete healing rate, *n* (%)	Follow‐up period
RAM flap	Collie *et al*.	2005	11	CD	11/11 (100)	3 months
	Cox *et al*.	1990	2	‐	2/2 (100)	3 months
RAM with HBOT	Chan	2014	4	Both	4/4 (100)	8–64 months
Gracilis muscle transposition	Collie *et al*.	2005	5	CD	1/5 (20)	3 months
	Maeda *et al*.	2011	4	CD	2/4 (50)	10 months
	Rius *et al*.	2000	7	CD	7/7 (100)	3–6 months
Omentoplasty	Yamamoto	2001	6	CD	5/6 (83)	23–32 months
Total excision of the sinus	Ferrari *et al*.	1980	3	CD	3/3 (100)	1.5 months

CD, Crohn's disease; HBOT, hyperbaric oxygen therapy; IBD, inflammatory bowel disease; RAM, rectus abdominis myocutaneous.

#### Surgical debridement, with or without closure

The first step in the treatment of a chronic wound is to debride fibrotic tissue, turning it into an acute wound. This can be done by opening the tract wide so that dressings and/or irrigation can be applied to the entire tract and not just to its exit site. A sinus has the shape of a fishbowl, and surgery is often necessary to change the wound contour into a saucer, which will allow for improved local wound care, decreased surface bacteria, and enhanced healing over time. Before surgical manipulation, a CT scan with oral contrast is often important to identify the dimensions of the tract and the risk to other vital structures. A shorter sinus results in more effective outcomes and less risk to vital structures if debridement is entertained. Over‐aggressive debridement of deep sinuses from a perineal approach can lead to direct injury of vital structures such as the small bowel.

Ferrari *et al*. have discussed their experience in performing excision of extensive PPSs that were inaccessible to curettage, necessitating the need for a more “aggressive” management approach. Their “total excision” required that all fibrous scar tissue was excised from the sinus with removal of one or two sacral bodies. The case series included seven patients who underwent “total excision” of a PPS following proctocolectomy for IBD. Wounds were healed by the 10th postoperative day in six patients. The seventh patient suffered from a hematoma, which was evacuated, with healing achieved by 3 weeks post excision.^1^ Typically, radical excision of fibrotic tissue in this manner precludes primary closure and creates a wound that would be amenable to flap closure as described below.

#### Flap closure of the perineum.

1. Flaps from above requiring a full laparotomy: These include rectus abdominis myocutaneous (RAM) flap, vertical rectus abdominis flap (VRAM), oblique rectus abdominis myocutaneous flap (ORAM), and omental flaps.

Deep and long sinuses may be best treated with surgery that debrides the tract both from the perineum (below) and from the abdomen (above). Laparotomy allows the surgeon to clear the small bowel from the surgical dissection, and the dissection from below is necessary to excise scarred tissues. This above and below approach is the most effective way to treat a persistent perineal sinus. Another consideration is whether laparotomy will be performed in an open fashion or with minimally invasive technology (i.e. laparoscope, robot). In many cases, the extensive scarring seen with PPS renders bowel dissection very difficult, if not impossible, with a minimally invasive approach.

After debridement, the resulting large space within the bony confines of the pelvis is best filled with supple, well‐vascularized soft tissues. Flaps based on the deep inferior epigastric artery are readily available when a laparotomy is performed. The RAM flap was first used for PPS closure in 1984, followed by a few subsequent case reports of the technique in patients with IBD.^22^ The procedure requires dissection of the rectus abdominis sheath along its full length off the anterior and posterior sheaths.^23^ Cox *et al*. utilized this approach in two CD patients, reporting successful PPS closure at 7 and 10 days. In a more recent case series by Collie *et al*., 15 patients with CD required surgical management of PPS following proctectomy. Eleven of these patients underwent RAM flap and all had a healed perineum within 3 months of surgery. On the basis of the study results, the authors recommend the use of the RAM flap method despite limited efficacy data in IBD patients.^22^ Potential disadvantages to RAM flaps include weakness of the anterior abdominal wall leading to development of incisional hernias,^23^, ^24^ as well as more difficult subsequent placement and construction of a stoma on the opposite side of the abdomen.^9^, ^24^ A recent systematic review concerning PPS management reports an 84% healing rate for RAM flaps and 64% for gracilis muscle transposition flaps (see below).^25^ A disadvantage of the RAM flap is that the denervated muscle atrophies over time, permitting the small bowel to descend into the pelvic cavity. The weakness caused by muscle loss and the pain associated with surgery are not to be underestimated. Despite these disadvantages compared to other rectus‐based flaps, a RAM can be elevated and placed robotically without a skin incision in those instances where an open approach is not required.

Strategies to improve on the RAM muscle‐only flap include flap designs that limit muscle disturbance and instead carry subcutaneous dermis and fat. The dermis and fat do not atrophy over time and therefore exclude the small bowel from the pelvis effectively over time. These flap designs require a skin and fascial incision and therefore cannot be performed robotically. The vertical rectus skin paddle (VRAM) takes a portion of the rectus muscle as well as the perforators to the skin. Partial rectus muscle preservation makes the flap perfusion a bit less reliable but with the benefits of fewer hernias and a better abdominal wall. The resulting scar appears to be an off‐center midline laparotomy. The oblique rectus abdominis flap (ORAM) relies on the anatomic knowledge that periumbilical perforators send their blood preferentially in a direction toward the tip of the scapula along dermatomes. The ORAM flap limits the total amount of muscle harvested within the flap. In the extreme, the surgeon can leave all of the muscle intact and just harvest the perforating blood vessel as it enters the subcutaneous tissue.^26^ These flaps have potentially less robust blood flow. A poorly perfused flap could require a re‐laparotomy, and so the reconstructive surgeon needs to ensure that the flap is healthy at the time of closure.

Chan *et al*. describe their experience of utilizing a combined technique in which they applied preoperative hyperbaric oxygen therapy (HBOT) to four CD patients with extreme PPS who underwent RAM flap repair. The patient is placed in a chamber filled with pure oxygen, thus increasing the oxygenation of blood and improving the healing process. Studies have shown that HBOT has a therapeutic effect in the IBD patient by decreasing oxidation stress, controlling inflammation, and improving cell repair.^27^ The goal of HBOT in the PPS patient was to relieve hypoxia and decrease pro‐inflammatory cytokines within the PPS. The authors reported four patients with PPS who achieved complete wound healing within 3 months without related hospital readmission over a median of 35 months of follow‐up. One patient suffered from preoperative HBOT‐induced aural barotrauma that resolved spontaneously.^28^ HBOT is a potentially safe and effective operative aid, although further studies are required.

2 Omental flap

The omentum is an extremely useful tissue that can be mobilized to fill the bony pelvis after sinus debridement. Its primary benefit is that it does not cause abdominal wall weakness or hernias. Not all post‐surgery patients have an intact omentum that can be mobilized successfully off the bowel, liver, and stomach. Typically, the laparotomy incision is extended toward the xyphoid, and the omentum is mobilized off the transverse colon while sparing and not injuring the transverse mesocolon. Although the omentum can be pedicled either off the right or left gastroepiploic vessel, the right side vessel is larger and preferred. The omentum is divided along its left side to reach the outer curve of the stomach. The short gastric vessels are divided to mobilize the omentum off attachments to the stomach and duodenum to reach the right gastroepiploic vessel. The omentum is passed either along the right gutter or immediately under the abdominal wall to reach the bony pelvis where it is held in place with several sutures. Yamamato *et al*. performed excision and omentoplasty on six CD patients with post‐proctocolectomy PPS. Following sinus excision, the omentum is brought down to the perineum and carefully sutured along the pelvic inlet to both cover the sinus and exclude the small bowel. The presacral sinus healed completely in five patients, and in one patient the omentum became necrotic and infected 1 month after the procedure, resulting in a complex sinus.^29^ They recommend preserving the omentum at the time of proctocolectomy and utilizing it to fill the resultant pelvic cavity whenever possible. Finally, in those instances where minimally invasive techniques are used to perform the laparotomy, the omentum can be harvested.

3 Gracilis muscle transposition

The gracilis muscle contributes minor functionality to daily activities yet has an abundant vascular pedicle from the medial circumflex femoral artery that can provide adequate vascularization for wound healing. The gracilis muscle transposition flap was first described in 1975 and a number of subsequent reports have described its utility. Use of the gracilis muscle rather than the rectus abdominis for the management of PPS conserves potential ostomy sites and avoids incisional hernia complications. Gracilis flaps are commonly used from below for rectourethral fistulas. However, a chronic draining sinus after a proctocolectomy is potentially a much longer and extensive problem. As stated above, it is challenging for the surgeon to debride the superior‐most aspect of a sinus only from below. The perineal debridement combined with a pedicled gracilis muscle flap is often insufficient.^22^ Rius and colleagues describe their experience treating three CD patients suffering from PPS managed with gracilis transposition; complete healing was achieved in all patients within a 6‐month period.^30^ In the previously discussed study by Collie *et al*., 5 of 15 patients with CD underwent gracilis transposition for PPS, with complete healing reported in only 1 of the 5.^22^ A more recent case series by Maeda *et al*. reported complete wound healing in two of four CD patients with PPS.^31^ Although of limited size, the gracilis muscle flap has little downside and can aid in the healing of perineal skin.

## Conclusion

PPS following proctocolectomy or proctectomy in IBD patients present a relatively common challenge for both the medical/surgical teams and the patients, alike. Current management varies with no definitive gold standard of care. The appropriate choice of treatment is dictated by varied clinical scenarios based on the location, size, and depth of the sinus as well as the available resources and experience among medical/surgical/wound care specialists. Future creative approaches, including the potential role of HBOT, to wound healing will ultimately help “fill the void” in treating this frustrating postoperative complication.
